# Characterization of a Novel PQQ-Dependent Quinohemoprotein Pyranose Dehydrogenase from *Coprinopsis cinerea* Classified into Auxiliary Activities Family 12 in Carbohydrate-Active Enzymes

**DOI:** 10.1371/journal.pone.0115722

**Published:** 2015-02-13

**Authors:** Kouta Takeda, Hirotoshi Matsumura, Takuya Ishida, Masahiro Samejima, Hiroyuki Ohno, Makoto Yoshida, Kiyohiko Igarashi, Nobuhumi Nakamura

**Affiliations:** 1 Department of Biotechnology and Life Science, Tokyo University of Agriculture and Technology, Tokyo, Japan; 2 Department of Biomaterials Sciences, Graduate School of Agriculture and Life Sciences, The University of Tokyo, Tokyo, Japan; 3 Department of Environmental and Natural Resource Science, Tokyo University of Agriculture and Technology, Tokyo, Japan; INRA, FRANCE

## Abstract

The basidiomycete *Coprinopsis cinerea* contains a quinohemoprotein (*Cc*PDH named as *Cc*SDH in our previous paper), which is a new type of pyrroloquinoline-quinone (PQQ)-dependent pyranose dehydrogenase and is the first found among all eukaryotes. This enzyme has a three-domain structure consisting of an N-terminal heme *b* containing a cytochrome domain that is homologous to the cytochrome domain of cellobiose dehydrogenase (CDH; EC 1.1.99.18) from the wood-rotting basidiomycete *Phanerochaete chrysosporium*, a C-terminal family 1-type carbohydrate-binding module, and a novel central catalytic domain containing PQQ as a cofactor. Here, we describe the biochemical and electrochemical characterization of recombinant *Cc*PDH. UV-vis and resonance Raman spectroscopic studies clearly reveal characteristics of a 6-coordinated low-spin heme *b* in both the ferric and ferrous states, as well as intramolecular electron transfer from the PQQ to heme *b*. Moreover, the formal potential of the heme was evaluated to be 130 mV vs. NHE by cyclic voltammetry. These results indicate that the cytochrome domain of *Cc*PDH possesses similar biophysical properties to that in CDH. A comparison of the conformations of monosaccharides as substrates and the associated catalytic efficiency (*k*_cat_/*K*_m_) of CcPDH indicates that the enzyme prefers monosaccharides with equatorial C-2, C-3 hydroxyl groups and an axial C-4 hydroxyl group in the ^1^C_4_ chair conformation. Furthermore, a binding study shows a high binding affinity of *Cc*PDH for cellulose, suggesting that *Cc*PDH function is related to the enzymatic degradation of plant cell wall.

## Introduction

Cellulolytic fungi are known to produce many extracellular oxidoreductases, such as peroxidase and laccases, as well as cellulolytic enzymes [1–4]. Recently, we discovered a pyranose dehydrogenase (*Cc*PDH named as *Cc*SDH in our previous paper) that represents a new type of pyrroloquinoline quinone (PQQ) quinohemoprotein from the basidiomycete *Coprinopsis cinerea* (formally known as *Coprinus cinereus*) strain 5338. This *Cc*PDH is the first such protein found among all eukaryotes [5]. The amino acid sequence of *Cc*PDH indicates that the extracellular enzyme contains three domains: an N-terminal cytochrome domain, a C-terminal carbohydrate binding module (CBM), and a central PQQ domain.

The N-terminal cytochrome domain of *Cc*PDH has 32 to 42% identity with the cytochrome *b* domains of basidiomycete cellobiose dehydrogenases (CDHs). CDH is a major oxidoreductase secreted by many cellulolytic fungi during growth on cellulose, and the genes that code for CDHs have been identified in numerous cellulolytic fungi [6]. CDH is an extracellular flavohemoprotein that consists of a catalytic FAD containing dehydrogenase domain, a heme *b* containing a cytochrome domain, and a linker region connecting the two domains. CDH catalyzes the dehydrogenation of cellobiose and cello-oligosaccharides to their corresponding δ-lactones. The oxidation of cellobiose takes place in the FAD domain, subsequently followed by inter-domain electron transfer to the cytochrome domain [7]. The cytochrome domain of CDH has unique structural features that fold into an antiparallel ß-sandwich and contains a 6-coordinate low-spin *b*-type heme with unusual Met-His ligands [8]. Because the cytochrome domain has the ability to communicate electrically with an electrode, CDH exhibits efficient direct electron transfer (DET) on an electrode surface and is an attractive biocatalyst for use in enzymatic biosensors or biofuel cells [9, 10]. According to the carbohydrate-active enzyme database (CAZy; www.cazy.org), the FAD and cytochrome domains of CDH are classified in subfamily 1 of Auxiliary Activity family 3 (AA3_1) and AA8, respectively [11]. *Cc*PDH has an AA8 cytochrome domain but a cellulose-binding domain attached to a PQQ domain instead of an FAD domain.

Several known prokaryotic dehydrogenases contain PQQ as their prosthetic group and mainly catalyze the oxidation of a number of alcohols or aldose sugars in the periplasm of Gram-negative bacteria [12–14]. In these dehydrogenases, PQQ is tightly but not covalently bound to the active site, which contains a divalent metal ion such as calcium [15]. Some of these PQQ quinoproteins contain an additional one or more hemes and are called quinohemoproteins [16, 17]. The known quinohemoproteins containing PQQ or other quinone cofactors almost exclusively have *c*-type heme cofactors [17, 18]. There are two types of PQQ-dependent glucose dehydrogenases: a membrane-bound form (mGDH) which has been identified in a wide range of bacteria [19, 20] and a soluble form (sGDH) that has been well described in *Acinetobacter calcoaceticus* [21, 22]. In addition, soluble aldose sugar dehydrogenases (Asd) from *Escherichia coli* [23] and *Pyrobaculum aerophilum* [24] are PQQ quinoproteins that have been identified relatively recently as homologues of the *A*. *calcoaceticus* sGDH. These Asds have oxidation activity towards a broad range of aldose sugars, with a weak affinity for substrates. The central PQQ domain of *Cc*PDH has low identity with sGDH from *A*. *calcoaceticus* (16%). The putative homologues of the PQQ domain of *Cc*PDH are widely distributed in bacteria, archaea, amoebozoa, and fungi. These quinoproteins belong to a different phylogenetic category from the known PQQ-quinoprotein family [5]. Therefore, the PQQ domain of *Cc*PDH is currently identified as a new Auxiliary Activities family 12 (AA12) in the CAZy database.

Many carbohydrate active enzymes including hydrolytic and non-hydrolytic proteins contain one or more non-catalytic CBMs that have functions in recognizing and adhering to carbohydrates [25–27]. Based on amino acid sequence similarities and three-dimensional structures, CBMs are currently grouped into 71 families in the CAZy database [28]. The amino acid sequence of *Cc*PDH indicates the presence of a family 1 CBM (CBM1). The majority of cellulose-binding domains attached to fungal cellulolytic enzymes belong to CBM1. The main role of CBM1 in cellulolytic enzymes is to enhance the hydrolysis of crystalline cellulose by increasing the effective enzyme concentration on the cellulose surface [29, 30]. CBM1s consist of less than 40 residues and usually contain two disulfide bonds and three solvent-exposed aromatic amino acids as critical residues for binding. CBM1s form a flat hydrophobic surface for adsorption to the hydrophobic surface of crystalline cellulose [31–35]. The application of CBMs for development of new carbohydrate-recognition technologies, such as affinity chromatography, have previously been examined [36].

As noted above, *Cc*PDH is an attractive multi-domain oxidoreductase that has three unique domains with different backgrounds. Until now, *Cc*PDH had been overexpressed in the methylotrophic yeast *Pichia pastoris*, and initial characterization of the recombinant protein has demonstrated the PQQ-dependence of the enzyme reactivity for the first time in a eukaryotic enzyme. Despite its low amino acid sequence homology with known PQQ-binding enzymes, this enzyme binds PQQ strongly and shows oxidation activity towards some sugars. In this study, we thoroughly investigated the enzymatic and electrochemical properties of recombinant *Cc*PDH.

## Materials and Methods

### Materials

Pyrroloquinoline quinone and equine heart cytochrome *c* were purchased from Sigma-Aldrich Japan G. K. (Tokyo, Japan). 2-Keto-D-glucose was purchased from Santa Cruz Biotechnology, Inc. (Santa Cruz, CA, USA). l-fucose, l-lyxose, and l-xylose were purchased from Wako Pure Chemical Industries (Osaka, Japan). d-arabinose and l-mannose were purchased from Sigma-Aldrich Japan G. K. (Tokyo, Japan). l-galactose, d-talose, and l-rhamnose were purchased from Funakoshi Co., Ltd. (Tokyo, Japan). l-gulose and l-glucose were purchased from Tokyo Chemical Industry Co., Ltd. (Tokyo, Japan).

### Preparation of *Cc*PDH and DH_PDH_

Recombinant intact *Cc*PDH and PQQ containing dehydrogenase domain (DH_PDH_) of *Cc*PDH (DH_PDH_: residues 240–649) were heterologously expressed in the methylotrophic yeast *Pichia pastoris* and purified as described previously [5]. The enzyme represented as “*Cc*PDH” in this paper means the heterologously expressed wild type. The purity of *Cc*PDH was confirmed by SDS/PAGE and by absorption spectroscopy. The *Cc*PDH concentration in the solution was estimated from the absorbance at 420 nm (ε_420_ = 130 mM^-1^ cm^-1^) [5].

### UV-vis spectroscopy

Electronic absorption spectra of *Cc*PDH and DH_PDH_ were recorded at room temperature with a JASCO V-660 spectrophotometer (Tokyo, Japan). *Cc*PDH was diluted in 50 mM HEPES buffer, pH 7.0, containing 1 μM PQQ and 1 mM CaCl_2_. Ca^2+^ is ligated with PQQ and several amino acid side chain atoms and is essential for the formation of active holo-enzyme of quinoproteins. The reduced form of *Cc*PDH was prepared by addition of 1 mM ascorbic acid or l-fucose. Apo-DH_PDH_ was stored in 50 mM HEPES buffer, pH 7.0, containing 1 mM CaCl_2_. Holo-DH_PDH_ was obtained by addition of PQQ to apo-DH_PDH_ according to our previous report [37]. The reduced form of DH_PDH_ was prepared by addition of 1 mM l-fucose.

### Resonance Raman spectroscopy

Resonance Raman (RR) spectra were measured on a JASCO NRS-1000 spectrometer using a Kaiser Optical holographic notch-plus filter and a liquid N_2_-cooled CCD detector (Princeton instrument, Spec-10, Trenton, NJ, U.S.A.). Raman scattering was excited by the 413.1 nm emission from a Kr ion laser (Innova 90C-K, Coherent Inc., Santa Clara, CA, U.S.A.), and the incident powers were 1 mW. The spectra were obtained with data accumulated over 30 sec and a spectral resolution of 1.47 cm^-1^. The spectra were calibrated against indene and CCl_4_ as external standards. A two-point baseline was performed with GRAMS/386 software. All measurements were carried out in 50 mM HEPES buffer (pH 7.0) containing 1 μM PQQ and 1 mM CaCl_2_, at room temperature. The reduced form of *Cc*PDH was prepared by the addition of 1 mM ascorbic acid or l-fucose.

### Measurement of midpoint potential

Redox responses of heme in *Cc*PDH were analyzed by cyclic voltammetry (ALS Electrochemical Analyzer 702B, BAS Inc., Tokyo, Japan). The potential was determined by averaging the anodic and cathodic peak potentials. The electrochemical responses of *Cc*PDH were obtained by using a carbon nanoparticle-modified plastic-formed carbon as a working electrode, as described elsewhere [38]. An Ag/AgCl (3 M NaCl) reference electrode (209 mV vs. NHE) and a platinum wire were used as the reference and counter electrodes, respectively. All measurements were performed in 50 mM HEPES buffer (pH 7.0) containing 1 μM PQQ and 1 mM CaCl_2_ using a three-electrode cell under a nitrogen atmosphere at room temperature.

### Enzyme assays and kinetic procedure

The enzyme activities of *Cc*PDH were assayed using cytochrome *c* as an electron acceptor [5, 39, 40]. The assay was performed by photometrically monitoring the time-dependent reduction of cytochrome *c* at 550 nm (Δε_550_ = 17.5 mM^-1^ cm^-1^) at 30°C. To obtain the kinetic parameters of oxidation for l-galactose, d-talose, l-xylose, and l-glucose, the assay was performed with various concentrations of monosaccharides (5–200 mM for l-galactose, 2.5–200 mM for d-talose, 5–300 mM for l-xylose, and 10–400 mM for L-glucose) in 50 mM Tris buffer, pH 8.5 containing 1 μM PQQ and 1 mM CaCl_2_. To determine the Michaelis constant (*K*_m_) and turnover number (*k*_cat_), experimental data were fitted with the Michaelis-Menten equation with using Origin 8.1 (OriginLab, Inc., Northampton, MA, U.S.A.) The activity of DH_PDH_ was determined using a dye-linked assay with the artificial electron acceptors phenazine methosulfate (PMS) as a primary electron acceptor and 2,6-dichlorophenolindophenol (DCPIP), according to our previous report [37]. The effect of PQQ binding on the activity of DH_PDH_ toward l-fucose was measured in 50 mM MES buffer, pH 6.5, containing 0.1 mM DCPIP, 1 mM PMS, 1 mM CaCl_2_, 100 mM l-fucose, 50 nM DH_PDH_, and varying concentrations of PQQ (2.5–100 nM). The reaction rate was measured by following the decrease in the absorbance of DCPIP at 520 nm (Δε_520_ = 6.9 mM^-1^ cm^-1^) [41] at 30°C using UV-vis spectroscopy.

### Sequence analysis of CBM1 of *Cc*PDH

The amino acid sequences of CBM1s within carbohydrate-active enzymes were collected from the CAZy database. The CBM1 sequences and the *Cc*PDH sequence were subjected to multiple-alignment analysis using MAFFT (ver. 6.85) [42, 43], and the figure was prepared using ESPript ver 2.3 [44].

### Adsorption of *Cc*PDH on celluloses

Adsorption experiments were carried out in 50 mM HEPES buffer, pH 7.0, using crystalline cellulose from *Cladophora* sp. and phosphoric-acid-swollen cellulose (PASC) prepared from Avicel according to the method described previously [45] as an amorphous substrate. Various concentrations of *Cc*PDH (0.5–32 μM) were incubated with 0.1% w/v celluloses for 1 h at 30°C and then were centrifuged for 20 min at 16,100 g. The absorbance at 420 nm, which corresponded to the Soret band of a *b*-type heme (ε_420_ = 130 mM^-1^ cm^-1^), was measured with UV-vis spectroscopy to determine the concentration of free enzyme in the supernatants. The equilibrium dissociation constant (*K*_d_) and the maximum amount of adsorbed enzyme (*A*_max_) were estimated as described previously [46, 47].

## Results

### UV-visible spectroscopy of *Cc*PDH

**Fig 1:**
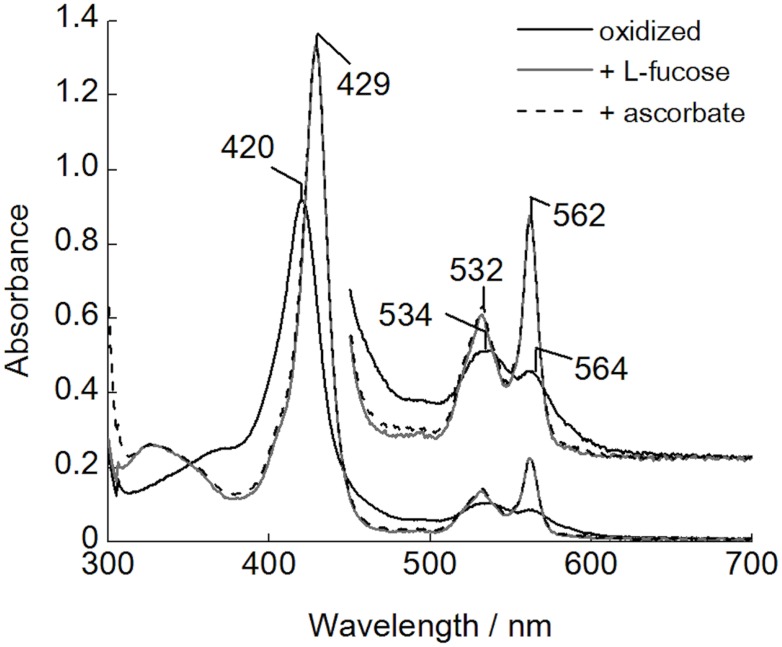
UV-visible absorption spectra of *Cc*PDH. Black solid line, oxidized form; gray solid line, reduced form by addition of L-fucose; dotted line, reduced form prepared by addition of ascorbic acid. All spectra were recorded in 50 mM HEPES buffer, pH 7.0, at room temperature.

The absorption spectra of the oxidized *Cc*PDH are shown in Fig. 1. The enzyme showed a typical hemoprotein spectrum with an absorption maximum at 420 nm and broad peaks at 534 nm and 564 nm, corresponding to the γ (Soret)-, β-, and α-bands, respectively. The reduction of *Cc*PDH by ascorbic acid, sodium dithionite, or l-fucose, which is a substrate, induced the same behavior, and no difference was observed in the resulting spectra. The absorbances of the γ-, β-, and α-bands were increased and the maxima of the bands shifted slightly to 429 nm, 532 nm, and 562 nm, respectively. The spectra shifts due to the reaction with l-fucose suggests that the electron generated by l-fucose can be transferred from the PQQ domain to the cytochrome domain, like that from the FAD domain to the cytochrome domain in the cellobiose oxidation of CDH.

### Resonance Raman spectroscopy

**Fig 2:**
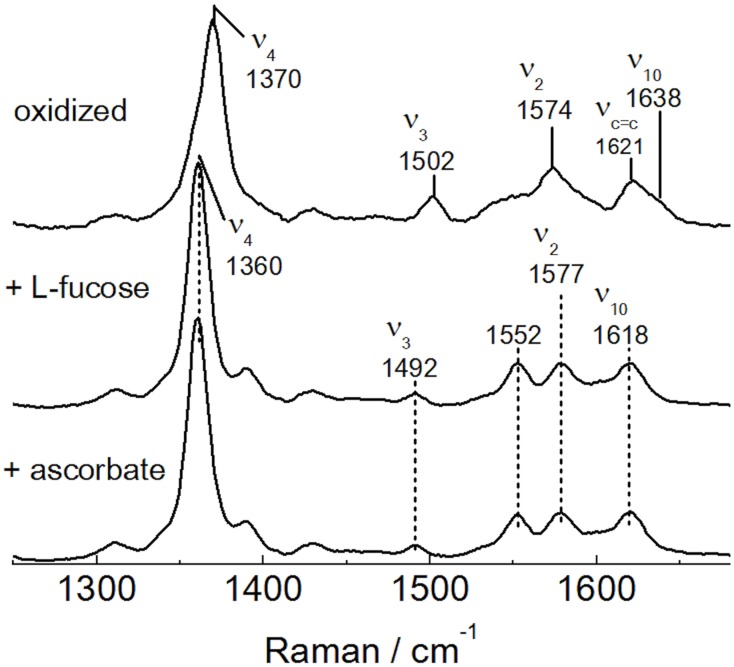
Resonance Raman spectra of the oxidized and reduced *Cc*PDH. From the top, oxidized form; reduced form prepared by addition of L-fucose; reduced form prepared with ascorbic acid. The measurements were carried out in 50 mM HEPES buffer, pH 7.0, at room temperature. The excitation wavelength and incident powers are 413.1 nm and 1 mW, respectively. All spectra were obtained with data accumulated over 30 sec with a spectral resolution of 1.47 cm^-1^.

Both the oxidized and reduced heme cofactors are RR active chromophores, and the vibrational modes can be enhanced with laser excitation in the 400–500 nm range. To further investigate the properties of the heme in *Cc*PDH, RR spectra were measured for both the oxidized and reduced forms of *Cc*PDH with excitation at 413 nm as shown in Fig. 2. The oxidation marker ν_4_ of *Cc*PDH in its oxidized form appears at 1370 cm^-1^, indicating a ferric heme. The core size marker bonds ν_2_ and ν_3_ of the oxidized form, occurring at 1574 and 1502 cm^-1^, respectively, identifies the ferric heme as having a hexacoordinate low-spin (6cls) heme state. The reduced form of *Cc*PDH prepared with either ascorbic acid or l-fucose showed the same RR spectral bands with the ν_2_, ν_3_, and ν_4_ to 1577, 1492, and 1360 cm^-1^, respectively. These values indicate that the reduced *Cc*PDHs have a ferrous heme with a 6cls state. The RR spectral data of the oxidized and the reduced forms of *Cc*PDH are summarized in Table 1 and compared with the cytochrome domain of CDH [48] from *P*. *chrysosporium*. The RR frequencies of oxidized and reduced *Cc*PDH were near agreement with those of CDH, suggesting that these cytochrome proteins have a similar microenvironment around the heme cofactor.

**Table 1:** Vibrational frequencies (cm^-1^) of *Cc*PDH and cytochrome domain of CDH.

Enzyme		ν_4_	ν_3_	ν_2_	ν_10_
*Cc*PDH	ferric	1370	1502	1574	1638
	ferrous	1360	1492	1577	1618
Cytochrome domain of CDH^*a*^	ferric	1371	1505	1575	1638
	ferrous	1362	1494	1580	1615

^*a*^ Ref. [48]

### Redox properties of *Cc*PDH

**Fig 3:**
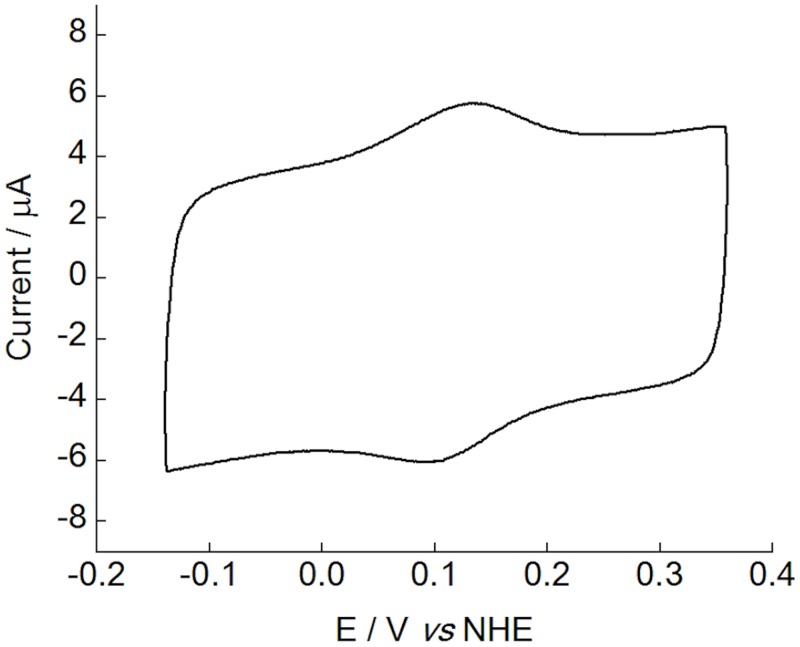
Cyclic voltammogram of *Cc*PDH immobilized on a plastic-formed carbon electrode modified with 27 nm carbon nanoparticles. The voltammogram was obtained in 100 mM HEPES buffer, pH 7.0, at a scan rate of 20 mV/s.

The reduction-oxidation (redox) potential of *Cc*PDH was estimated by cyclic voltammetry. As shown in Fig. 3, well-defined anodic and cathodic peaks can be observed, indicating a direct electron transfer between *Cc*PDH and the electrode. The redox potential determined from the midpoint of the oxidation-reduction peak potentials is 130 mV vs. NHE at pH 7. This is in accordance with previous reports describing the cytochrome domain of CDH from *P*. *chrysosporium* in solution (130 mV, pH 7) [39]. Thus, this redox potential was assigned to the Fe^3+^/Fe^2+^ redox couple of the heme *b* center in *Cc*PDH.

### Absorption spectra and PQQ dependent activity of DH_PDH_

**Fig 4:**
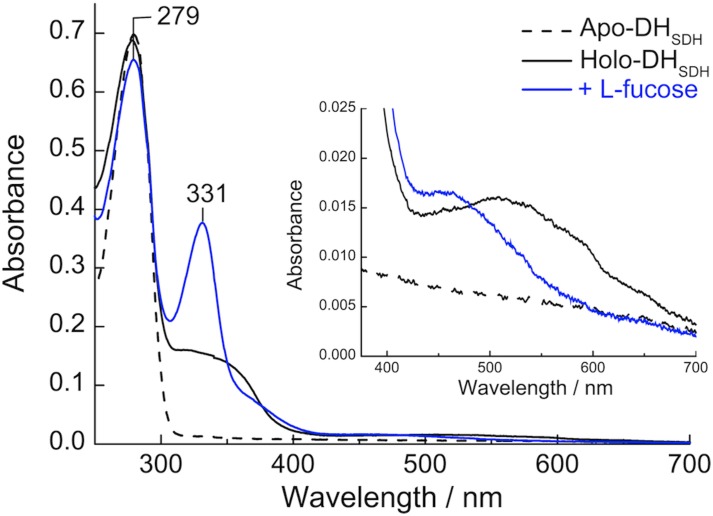
UV-visible absorption spectra of the apo- and holo-forms of DH_PDH_. Dotted line, apo-form of DH_PDH_; black solid line, holo-form of DH_PDH_; blue solid line, reduced form by addition of 1 mM l-fucose. All spectra were recorded in 50 mM HEPES buffer, pH 7.0 at room temperature.

To extend the studies of PQQ binding to *Cc*PDH, we used DH_PDH_ that was separated from a cytochrome domain and a CBM1 by a linker region. The UV-visible spectrum of the purified DH_PDH_ was typical of an enzyme without a cofactor. It had an absorbance maximum at 279 nm with aromatic amino acid residues (Fig. 4). Upon reconstitution with PQQ, the absorption spectrum of holo-DH_PDH_ showed a peak with a maximum at 279 nm, a broad band at around 340 nm with a shoulder at 365 nm, and a less intense and broad band from 420 to 700 nm, centered at around 510 nm. These absorption bands, which overlapped with the α-, β-, and γ-band from cytochrome domain of *Cc*PDH, resulted from using DH_PDH_. The addition of l-fucose resulted in the reduction of PQQ and the formation of a sharp absorption band at 331 nm; additionally, the maximum of the low broad band shifted from 510 nm to 460 nm. These results were similar to those reported for the quinoprotein sGDH from *A*. *calcoaceticus* [15, 21].

**Fig 5:**
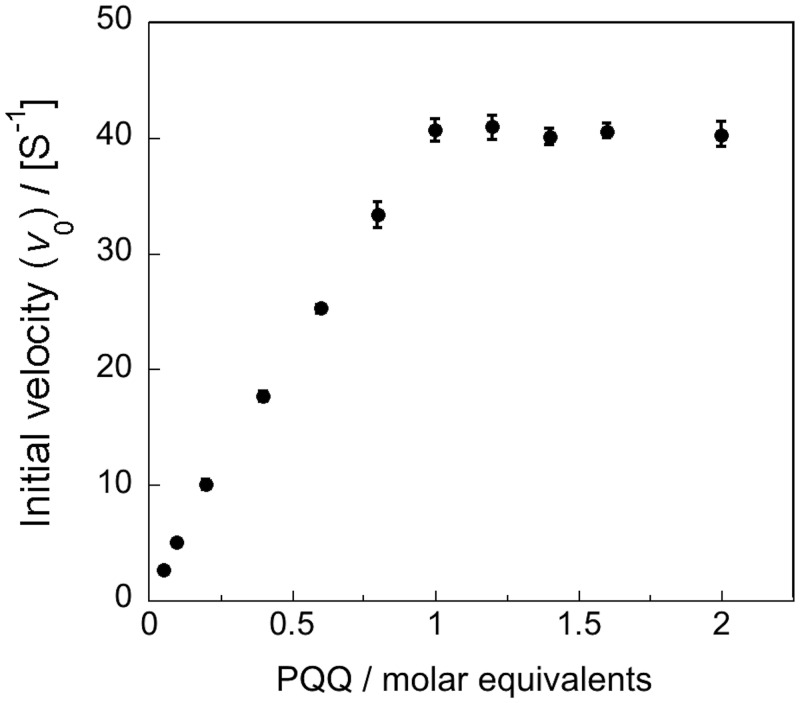
Titration of the apo-form of DH_PDH_ with PQQ. The purified apo-form of DH_PDH_ (50 nM) was pre-incubated with various concentrations of PQQ in 50 mM MES buffer, pH 6.5, containing 1 mM CaCl_2_. After 1 min, the enzyme activity was determined according to the procedure described in the Experimental Procedures.

Fig. 5 shows that the catalytic activity of DH_PDH_ increased with stoichiometric amounts of PQQ. It was estimated that full activity was attained at a molar ratio (PQQ: DH_PDH_) of 0.99. This value increases in linear manner to the equivalence point of the PQQ, indicating that the affinity of apo-DH_PDH_ for PQQ is high, similar to bacterial PQQ quinoproteins [21, 23, 49, 50]. Previous experiments using isothermal titration calorimetry (ITC) have shown that DH_PDH_ binds PQQ with a 1:1 stoichiometry and has strong affinity with a dissociation constant (*K*_d_) of 1.11 nM [5]. These results clearly indicate that *Cc*PDH is a PQQ-dependent dehydrogenase.

### Catalytic properties of *Cc*PDH

Our previous studies suggest that *Cc*PDH catalyzes the dehydrogenation of monosaccharides having a ^1^C_4_ chair conformation, such as l-fucose and d-arabinose [5]. We examined the catalytic activity toward the additional monosaccharides l-galactose, d-talose, l-xylose, l-glucose, l-lyxose, l-mannose, and l-rhamnose (= 6-deoxy- L-mannose), noting the configuration of the substrate as shown in Table 2. The *k*_cat_ value towards l-galactose was comparable to l-fucose, which is a 6-deoxy sugar of l-galactose, whereas the *K*_m_ values of l-galactose were twice that of l-fucose. The affinity for d-talose was about the same as for l-fucose, but the *k*_cat_ value was decreased by 30%. The *K*_m_ values for l-xylose and l-glucose were approximately 236 mM and 363 mM, respectively, which are markedly greater than the *K*_m_ obtained for the former monosaccharides. Slight oxidation activities of *Cc*PDH were observed for l-lyxose and l-mannose, but little was observed for l-rhamnose. Thus, the catalytic efficiency was highest for d-glucosone, followed by l-fucose > d-arabinose ≈ l-galactose > d-talose ≈ l-gulose > d-lyxose > l-xylose ≈ l-glucose.

**Table 2:** Specificity constant values of *Cc*PDH for various monosaccharides.

	Side group orientations in the ^1^ *C*_4_ conformation^*a*^				Kinetic parameters			
	C-2	C-3	C-4	C-5	*k*_cat_ (s^-1^)	*K*_m_ (mM)	*k*_cat_/*K*_m_(×10^3^ s^-1^M^-1^)	Relative velocity^*c*^
d-glucosone^*b*^	E (C-3)	E (C-4)	A (C-5)	- (C-6)	74.1 (±1.4)	7.9 (±0.3)	9.37	100
l-fucose^*b*^	E	E	A	E (-CH_3_)	56.4 (±1.8)	24.8 (±1.2)	2.27	77.7
d-arabinose^*b*^	E	E	A	-	35.5 (±0.6)	30.3 (±0.8)	1.17	47.5
l-galactose	E	E	A	E (-CH_2_OH)	56.1(±1.1)	49.7 (±1.4)	1.13	65.4
d-talose	E	A	E	A (-CH_2_OH)	17.8 (±0.1)	23.1 (±0.4)	0.77	24.8
l-gulose^*b*^	E	A	A	E (-CH_2_OH)	53.3 (±1.1)	84.7 (±2.6)	0.63	52.1
d-lyxose^*b*^	E	A	A	-	12.9 (±0.3)	66.8 (±2.6)	0.19	13.4
l-xylose	E	E	E	-	22.4 (±1.2)	236 (±16)	0.09	11.9
l-glucose	E	E	E	E (-CH_2_OH)	24.9 (±1.2)	363 (±17)	0.07	9.2
l-lyxose	A	E	E	-	n.d.^*d*^	n.d.	-	4.0
l-mannose	A	E	E	E (-CH_2_OH)	n.d.	n.d.	-	3.6
l-rhamnose	A	E	E	E (-CH_3_)	n.d.	n.d.	-	0.7

^*a*^ Carbon numbers of d-glucosone in brackets; E, equatorial bond; A, axial bond.

^*b*^ Data for the kinetic parameters are taken from Ref. (5).

^*c*^ The velocity with each substrate is expressed relative to 100 for the velocity with d-glucosone. The initial substrate concentration was 30 mM d-glucosone and 100 mM the others monosaccharides.

^*d*^ n.d., not determined.

### Carbohydrate-binding properties of *Cc*PDH

**Fig 6:**
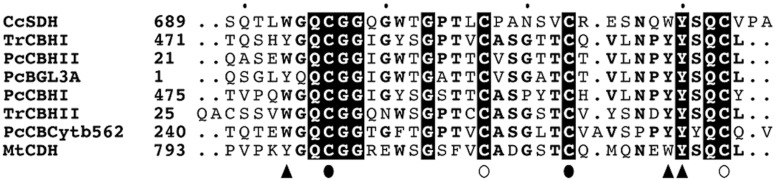
Multiple alignments of the amino acid sequences of CBM1 of *Cc*PDH and other known CBM1s. Residues in bold are highly conserved and those in boxes with a black background are perfect matches. Aromatic residues that are candidates for carbohydrate binding are indicated by a filled arrow, and two pairs of cysteines forming disulfide bonds are indicated by filled and open circles, respectively. *Tr*CBHI, cellobiohydrolase I (Cel7A) from *Trichoderma reesei* (accession no. P62694); *Pc*CBHII, cellobiohydrolase II (Cel6A) from *Phanerochaete chrysosporium* (Q02321); *Pc*BGL3A, glucan β-1,3-glucosidase (Bgl) from *P*. *chrysosporium* (Q8TGC6); *Pc*CBHI, cellobiohydrolase I-2 (Cel7D) from *P*. *chrysosporium* (Q09431); *Tr*CBHII, cellobiohydrolase II (Cel6A) from *T*. *reesei* (P07987); *Pc*CBCytb562, carbohydrate-binding cytochrome *b*_562_ from *P*. *chrysosporium* (Q66NB8); *Mt*CDH, cellobiose dehydrogenase from *Myceliophthora thermophila* (O74240).

**Fig 7:**
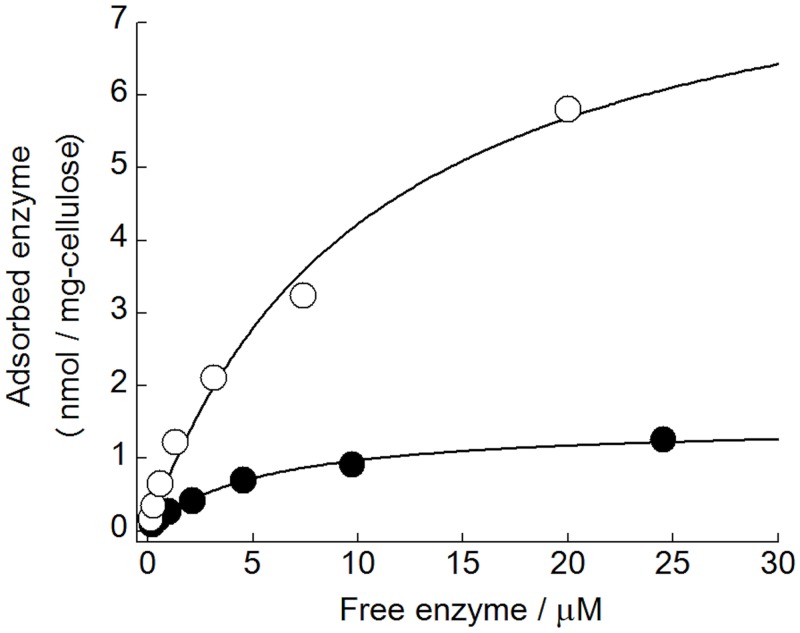
Enzyme concentration dependence of the amount of adsorbed *Cc*PDH. Closed circle, highly crystalline cellulose from *Cladophora*; open circle, PASC. The adsorption of *Cc*PDH was measured after incubation for 120 min with 1 mg/mL of cellulose at 30°C as described in the Experimental Procedures.

The amino acid sequence of *Cc*PDH indicated the presence of a CBM Family 1 C-terminal domain of *Cc*PDH. The CBM1 homologous sequence of *Cc*PDH was aligned with other CBM1s from fungal cellobiohydrolases (CBHs), ß-glucosidase (BGL), carbohydrate-binding cytochrome *b*_562_ (CBCyt. *b*_562_) and the basidiomycete CDH using MAFFT multiple alignment, as shown in Fig. 6. The multiple sequence alignment shows that the C-terminal CBM1 of *Cc*PDH contains three conserved aromatic residues (Trp693, Trp719, and Tyr720) that are proposed to contribute to *Cc*PDH binding on the surface of carbohydrates. It also shows conserved cysteines (Cys696, 707, 713, and 723) involved in disulfide bond formation. The amount of *Cc*PDH adsorbed on insoluble cellulose was measured at various protein concentrations after incubation for 2 hours. A highly crystalline cellulose I_a_ from *Cladophora* and a PASC, which is an amorphous cellulose obtained by phosphoric acid treatment, were used as carbohydrate materials. Fig. 7 shows the plot of the enzyme absorbed on cellulose versus free enzyme concentration. The calculated values for *A*_max_ and *K*_*d*_ from the corresponding plots are shown in Table 3. The *A*_max_ on PASC showed higher adsorption by *Cc*PDH than on cellulose I_a_ from *Cladophora* and the binding efficiency (*A*_max_/*K*_*d*_) for PASC was approximately 11 times higher than that for cellulose I_a_. This is likely due to the difference in the surface area where the enzyme can adsorb. Therefore, the results suggest *Cc*PDH is a redox enzyme that has the ability to adsorb on cellulose surfaces as well as CDHs.

**Table 3:** Adsorption parameters of *Cc*PDH for highly crystalline celluloses from *Cladophora* and PASC.

	*K*_d_ (μM)	*A*_max_ (nmol/mg-cellulose)	*A*_max_ / *K*_d_
Crystalline celluloses	0.19	1.48	7.79
PASC	0.10	8.69	86.9

The parameters were derived from adsorption data plotted as described in Experimental Procedures.

## Discussion

In a previous homology search with the cytochrome domain of CDH from *P*. *chrysosporium*, *Cc*PDH was discovered in the *C*. *cinerea* 5338 strain as a new quinohemoprotein. *Cc*PDH has a three-domain organization, with a catalytic domain containing PQQ as a cofactor (DH_PDH_), a *b*-type cytochrome domain as a redox site, and a CBM1. As far as we know, this is the first example of an extracellular quinohemoprotein connected to a cytochrome domain of CDH and CBM1. In this study, the spectroscopic and electrochemical properties, substrate specificity, and carbohydrate-binding properties of this novel PQQ-dependent enzyme from *C*. *cinerea* were determined.

The N-terminal cytochrome domain of *Cc*PDH shows significant homology to that of CDH, with well-conserved Met/His ligands for heme binding and a disulfide bond. The structural homology modeling of the cytochrome domain of *Cc*PDH by the Phyre program provided structural folds into an immunoglobulin-like ß-sandwich consisting of a five-stranded and a six-stranded ß-sheet with a short α-helical structure at the C-terminus, which is an unusual module among cytochromes [5]. The electronic absorption and RR spectra of *Cc*PDH in the oxidized and reduced forms are in good agreement with those previously reported for the cytochrome domain of CDH from *P*. *chrysosporium*. Those studies confirm a 6cls heme *b* in both the ferric and ferrous states with Met/His ligands, like CDH. In addition to spectroscopic results, electrochemical studies indicate that the redox potential of the heme is +130 mV, which is comparable to CDH from *P*. *chrysosporium*. These observations suggest that the cytochrome domain of *Cc*PDH may have a similar electron transfer function to the cytochrome domain of CDH, i.e., intra- and inter-molecular electron transfer between the cytochrome domain and the catalytic domain and also Fe (III)-reducing ability. Indeed, the electron transfer between *Cc*PDH and cytochrome *c* can be observed, as it is commonly used as an electron acceptor in the CDH reaction. CDHs are well known as redox enzymes capable of DET between the enzyme and the electrode and the cytochrome domain can act as a built-in electron-transfer mediator [9, 10, 51]. The cytochrome domain of *Cc*PDH was also capable of DET. Thus, *Cc*PDH is an excellent candidate for construction of DET-system bioelectronic devices such as biosensors and biofuel cell anodes, as well as CDHs.

Interestingly, *Cc*PDH exhibited dehydrogenase activity towards monosaccharides in a ^1^C_4_ chair conformation, in contrast to the substrate specificity of *A*. *calcoaceticus* sGDH, which has an absolute preference for l-arabinose and d-xylose in the ^4^C_1_ conformation over the d-arabinose and l-xylose enantiomers [52]. The membrane-bound quinoprotein GDH from *E*. *coli* also lacks activity for l-hexoses or pentoses that have a ^1^C_4_ chair conformation, such as l-glucose, l-mannose, l-rhamnose, and l-xylose and d-pentoses [53]. Additionally, Asd from *E*. *coli* exhibited no preference for different enantiomers with d- and l-arabinose [23].

**Fig 8:**
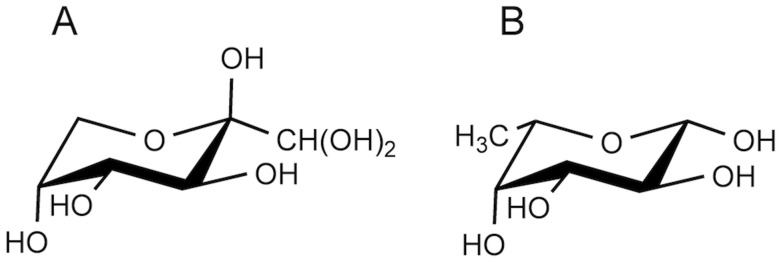
Structure of d-glucosone (A) and l-fucose (B) in a ^1^C_4_ conformation.

The compared catalytic activity of *Cc*PDH for these substrates and the side group orientation of each monosaccharide when in a ^1^C_4_ conformation are summarized in Table 2 and shown for d-glucosone and l-fucose in Fig. 8. d-glucosone (= 2-keto-D-glucose) has four tautomer forms reported by Freimund et al [54]. The ^1^C_4_ conformation of 2-keto-D-glucopyranose is the one of the tautomer, at 21% abundance in aqueous solution, as shown by Fig. 8A. The carbons are numbered from the position of the oxygen atom that forms part of the ring. Because these atoms are shifted, the apparent carbon positions of the side group orientations of d-glucosone are shown with the corresponding carbon number of the other monosaccharides in a ^1^C_4_ conformation (Table 2). The results shown in Table 2 reveal insights into the substrate recognition dependence on the active site of the catalytic domain in *Cc*PDH. This enzyme showed high activity for aldose substrates with C-2 and C-3 hydroxyl groups in an equatorial configuration and C-4 hydroxyl groups in axial configurations, such as l-fucose, d-arabinose, and l-galactose. In light of these results, we inferred that *Cc*PDH recognizes a ^1^C_4_ conformation of d-glucosone (Fig. 8A). The equatorial C-2 hydroxyl group of substrate is essential for binding to the active site of *Cc*PDH, as indicated by a markedly decreased activity for l-lyxose, l-mannose and no activity for l-rhamnose, which have axial C-2 hydroxyl groups. The axial C-4 hydroxyl group is also important for recognition; the *K*_m_ value increased by an order of magnitude when it the C-4 hydroxyl was replaced with an equatorial bond (in l-xylose and l-glucose compared to d-arabinose and l-galactose). The *K*_m_ value for l-gulose, which is a C-3 epimer of l-galactose, was approximately 1.7-fold higher than that of l-galactose. This value indicates that the C-3 hydroxyl group in the equatorial conformation is preferred over the axial for *Cc*PDH. The affinity for pentose sugars, which lack a hydroxymethyl group at the C-5 position, or a 6-deoxy-hexose, which has methyl group at C-5 (in L-fucose), increased over hexose sugars that have a C-5 hydroxymethyl group, suggesting that the hydroxymethyl group at this position causes some steric hindrance. However, the result suggests that C-5 methyl group is not critical for binding to *Cc*PDH, as indicated by the oxidation of l-fucose. In light of the stereospecificity of other PQQ-dependent dehydrogenases [20, 52, 55], *Cc*PDH may catalyze the dehydrogenation reaction of regioselective substrates at the C-1 hydroxyl group, although we can not rule out side reactions at other positions. Further studies are necessary to identify a product of *Cc*PDH oxidation and they are currently under consideration.

Many extracellular fungal carbohydrate hydrolytic enzymes carry a CBM1, which is thought to facilitate binding to the surface of cellulose chains and is related to the catalytic function of these enzymes. Although these CBM1 are not found in basidiomycetous CDHs, the flavocytochrome enzymes are also capable of binding to cellulose by its flavin domain and localizes on cellulose surfaces [56–58]. In the present study, *Cc*PDH shows high affinity toward microcrystalline and amorphous cellulose. This result suggests the possibility of localizing *Cc*PDH on the surface of cellulose, as is the case for CDHs. Further cytochemical analysis will be needed to identify the localization of *Cc*PDH in nature.

Under aerobic conditions, cellulose degradation by cell-free culture filtrates of various fungi has shown much higher rates compared to anaerobic conditions, suggesting that an oxidative reaction may play a crucial role in the fungal degradation of cellulose. CDH is the first example of an oxidoreductase related to the extracellular oxidative process for cellulose degradation. Furthermore, it has been recently reported that the cytochrome domain of CDH can transfer electrons to fungal copper-dependent lytic polysaccharide monooxygenases (LPMOs) that were classified in AA9. The combination of CDHs and LPMOs synergistically enhances cellulase activity [59–62]. The cytochrome domain of *Cc*PDH could allow direct electron transfer for LPMO as well as that of CDH. However the physiological functions remain obscure *in vivo*. It is not only some redox proteins instead of CDH, but some reductive compounds are the possibly electron donor for LPMO in those fungi. Indeed, l-ascorbic acid is typically used as an electron donor for LPMO *in vitro* [63]. The oxidation product of d-glucosone by *Cc*PDH could be an intermediate in the l-ascorbic acid biosynthesis [64]. PQQ quinoproteins also play a major role in the bacterial production of l-ascorbic acid [55, 65, 66]. Fugal *Penicillium cyaneo-fulvum* has an extracellular d-erythorbic acid (isoascorbic acid) synthesis pathway by using glucose oxidase and gluconolactone oxidase [67]. *Cc*PDH, therefore, may play a role in the l-ascorbic acid biosynthesis pathway and then LPMOs could be provided with electrons from these reductive compounds. Anyhow, *Cc*PDH must be related to the enzymatic degradation of the plant cell wall because of its capability of binding to cellulose.

## Conclusions

We have demonstrated the full characterization of *Cc*PDH, which was classified as a member of the new AA12 family in CAZy using spectroscopic and electrochemical methods. These enzymatic properties provide new insight into other known PQQ quinoproteins as well as the basidiomycetous oxidoreductase network. The N-terminal cytochrome domain of *Cc*PDH contains a 6-coordinated low-spin heme *b* in both the ferric and ferrous states with Met/His ligands as well as CDH and it enables DET between *Cc*PDH and the electrode. The catalytic domain shows oxidation activity toward monosaccharides in a ^1^C_4_ chair conformation in the presence of PQQ as a cofactor, and the most efficient catalysis was observed for d-glucosone. *Cc*PDH exhibited binding affinity for insoluble cellulose. These results suggest that *Cc*PDH may be involved in the extracellular oxidative degradation as well as CDH. Therefore, these proteins are an attractive component of cellulolytic enzymes for use in biodegradation and biomass conversion or as an anode catalyst for bioelectrochemical applications.
